# Isolated Adrenocorticotropin Deficiency With Depressive Symptoms

**DOI:** 10.1210/jcemcr/luaf304

**Published:** 2026-02-24

**Authors:** Kentaro Mikami, Yasushi Oiwa, Mitsuhiro Kometani, Takashi Yoneda, Masashi Oe, Yuko Katsuda

**Affiliations:** Department of Diabetes and Endocrinology, Fukui Prefectural Hospital, Fukui, Fukui 910-8526, Japan; Department of Health Promotion and Medicine of Future, Kanazawa University Graduate School of Medicine, Kanazawa, Ishikawa 920-8641, Japan; Department of Health Promotion and Medicine of Future, Kanazawa University Graduate School of Medicine, Kanazawa, Ishikawa 920-8641, Japan; Department of Health Promotion and Medicine of Future, Kanazawa University Graduate School of Medicine, Kanazawa, Ishikawa 920-8641, Japan; Department of Diabetes and Endocrinology, Fukui Prefectural Hospital, Fukui, Fukui 910-8526, Japan; Department of Diabetes and Endocrinology, Fukui Prefectural Hospital, Fukui, Fukui 910-8526, Japan

**Keywords:** isolated ACTH deficiency, adrenal insufficiency, adrenocorticotropin, depression, hydrocortisone

## Abstract

Isolated adrenocorticotropin deficiency (IAD) is a rare cause of adrenal insufficiency, often presenting with nonspecific symptoms that can be misattributed to mental disorders or gastrointestinal diseases, particularly in older adults. IAD is characterized by profoundly reduced adrenocorticotropin (ACTH) secretion with preserved secretion of other anterior pituitary hormones. We describe an 81-year-old woman with persistent nausea, vomiting, anorexia, and depressive symptoms that did not respond to standard antidepressant agents. During 2 months of hospitalization, she progressed to impaired consciousness. Routine laboratory tests and magnetic resonance imaging of the pituitary region revealed no abnormalities. Hormonal evaluation showed markedly low levels of cortisol and ACTH. A corticotropin-releasing hormone stimulation test confirmed IAD. The patient had no history of glucocorticoid use, immune checkpoint inhibitors therapy, or other causes of adrenal insufficiency. Intravenous hydrocortisone replacement therapy at 50 mg daily led to a rapid improvement in consciousness and resolution of psychiatric gastrointestinal symptoms. This case emphasizes the importance of considering IAD in older patients presenting with unexplained neuropsychiatric and gastrointestinal symptoms, particularly when standard therapies are ineffective. Early diagnosis and hormone replacement therapy are critical as delayed recognition may lead to life-threatening deterioration, but the condition remains reversible with appropriate management.

## Introduction

Isolated adrenocorticotropin (ACTH) deficiency (IAD) is a rare endocrine disorder characterized by selective ACTH deficiency with preserved secretion of other pituitary hormones [1]. Reduced ACTH levels result in insufficient cortisol secretion, leading to life-threatening conditions.

Acquired IAD is associated with glucocorticoid use, autoimmunity, cancer treatment with immune checkpoint inhibitors, pituitary trauma, Sheehan syndrome, empty sella syndrome, and radiation therapy [2]. Although the prevalence of IAD remains unknown, its recognition has increased over the last decade, particularly in patients receiving immune checkpoint inhibitors [2].

The clinical presentation of IAD is nonspecific, making its diagnosis challenging. Symptoms such as fatigue, loss of appetite, weight loss, nausea, vomiting, and abdominal pain can mimic neuropsychiatric or gastrointestinal disorders [1, 3].

Diagnosis is confirmed by demonstrating low cortisol levels with inappropriately low ACTH levels, along with an inadequate cortisol response to ACTH stimulation testing and an inadequate ACTH response to corticotropin-releasing hormone (CRH) stimulation. Hydrocortisone replacement therapy is the standard treatment for IAD [1, 3].

Here, we present the case of an 81-year-old woman with persistent nausea, vomiting, weight loss, and depressive symptoms who was ultimately diagnosed with IAD.

## Case Presentation

An 81-year-old woman was admitted to the psychiatry department of our hospital with complaints of chronic nausea, vomiting, and depressive symptoms.

The patient had been in a normal state of health until 2 months before admission, when she noticed nausea. One month before admission, the patient presented with amnesia, which was evaluated by a local physician. Computed tomography (CT) of the head showed no abnormalities.

Two weeks before admission, she presented to the emergency department of our hospital for persistent nausea and vomiting and was subsequently referred to the psychiatry department. At that time, she presented with a decline in interest and motivation, which led her to refrain from going out. Findings on physical examination, electrocardiography, chest and abdominal CT, magnetic resonance imaging (MRI) of the brain, and upper gastrointestinal endoscopy were unremarkable. Because a psychological cause was suspected, she was admitted to the psychiatry department of our hospital.

On admission, her body temperature was 36.1 °C, pulse rate was 91 beats/min, and blood pressure was 104/62 mm Hg. Her height, weight, and body mass index were 151.8 cm, 61.8 kg, and 26.8, respectively. Oxygen saturation was 94%. The patient remained awake, alert, and oriented. Her head, neck, chest, and abdomen were normal. There was no skin hyperpigmentation or peripheral edema. Routine hematological and biochemical parameters were within normal limits. Electrolyte and endocrinological values are summarized in Table 1. As her depressive symptoms met the diagnostic criteria for major depressive disorder, mirtazapine therapy was initiated. Two weeks after hospitalization, olanzapine was added, and 3 weeks after hospitalization, duloxetine was introduced. Despite these interventions, nausea and vomiting persisted, and her appetite continued to decline.

**Table 1.** Laboratory data

Variable	Reference range	5 d before admission	8 wk after hospitalization
Sodium	138.0-145.0 mmol/L	142.6 mmol/L	137.8 mmol/L
Potassium	3.60-4.80 mmol/L	3.79 mmol/L	4.01 mmol/L
Chloride	101.0-108.0 mmol/L	108.1 mmol/L	96.9 mmol/L
Calcium	8.8-10.1 mg/dL (SI: 2.20-2.52 mmol/L)	9.7 mg/dL (SI: 2.42 mmol/L)	10.0 mg/dL (SI: 2.50 mmol/L)
Free thyroxine	0.76-1.65 ng/dL (SI: 9.8-21.2 pmol/L)	0.83 ng/dL (SI: 10.7 pmol/L)	0.71 ng/dL (SI: 9.1 pmol/L)
Free triiodothyronine	2.39-4.06 pg/mL (SI: 3.7-6.2 pmol/L)	2.91 pg/mL (SI: 4.5 pmol/L)	3.36 pg/mL (SI: 5.2 pmol/L)
Thyrotropin	0.61-4.23 µIU/mL	2.043 µIU/mL	5.126 µIU/mL
Cortisol	7.07-19.60 µg/dL (SI: 195-541 nmol/L)	ND	0.37 µg/dL (SI: 10.2 nmol/L)
Adrenocorticotropin	7.2-63.3 pg/mL	ND	< 1.5 pg/mL
Prolactin	3.12-15.39 ng/mL	ND	34 ng/mL
Insulinlike growth factor I	43-149 ng/mL (SI: 5.6-19.5 nmol/L)	ND	55 ng/mL (SI: 7.2 nmol/L)
Growth hormone	0.13-9.88 ng/mL	ND	0.26 ng/mL
Luteinizing hormone	5.72-64.31 mIU/mL	ND	20.71 mIU/mL
Follicle-stimulating hormone	≤157.79 mIU/mL	ND	39.34 mIU/mL

Reference ranges for prolactin, luteinizing hormone, and follicle-stimulating hormone were based on values established for postmenopausal women. The reference range for insulinlike growth factor I was based on values established for females aged 80 years or older.

Abbreviations: ND, no data; SI, international system of units.

**Figure 1.**
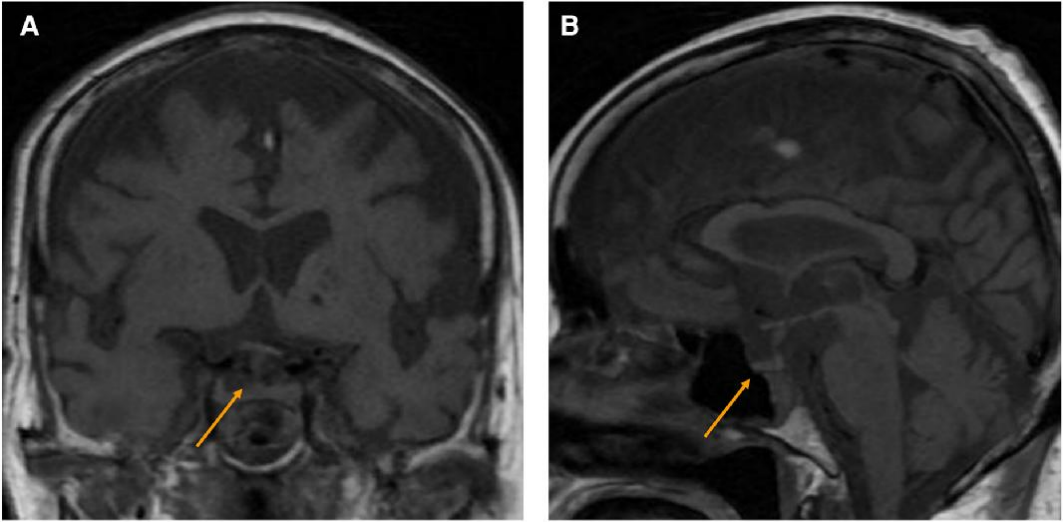
Magnetic resonance imaging findings of the sella turcica obtained 8 weeks after hospitalization. A, Sagittal and B, coronal T1-weighted images obtained 8 weeks after hospitalization showed no abnormalities in the pituitary gland.

Seven weeks after hospitalization, owing to the decreased level of consciousness, the patient failed to respond to commands and total parenteral nutrition was initiated. Eight weeks after hospitalization, MRI of the sella turcica was unremarkable (Fig. 1). Laboratory tests revealed markedly low cortisol (0.37 µg/dL [SI: 10.2 nmol/L]; reference range, ≥ 18 µg/dL [SI: ≥ 497 nmol/L]) and ACTH (< 1.5 pg/mL; reference range, 7.2-63.3 pg/mL) levels. The sodium level was slightly below the reference range at 137.8 mmol/L (reference range, 138.0-145.0 mmol/L). Prolactin, luteinizing hormone, and follicle-stimulating hormone levels were within the reference ranges for postmenopausal women (see Table 1).

Nine weeks after hospitalization, she was referred to the endocrinology department of our hospital for further assessment.

## Diagnostic Assessment

The patient's medical history included hypertension, hyperlipidemia, uterine leiomyoma, and a previous occurrence of appendicitis. She had no history of chronic glucocorticoid use, autoimmune disease, tuberculosis, trauma, or cancer immunotherapy. There was no family history of endocrine disorders, and she did not smoke or drink alcohol. Secondary adrenal insufficiency was suspected, and the patient underwent dynamic testing.

An adrenocorticotropin stimulation test was performed. This test evaluates primary adrenal insufficiency by measuring cortisol levels before and 30 and 60 minutes after a 250-mcg bolus intravenous (IV) injection of adrenocorticotropin. The peak stimulated serum cortisol level was low at 3.46 µg/dL (SI: 95.5 nmol/L) at 60 minutes (reference range, ≥ 18 µg/dL [SI: ≥ 497 nmol/L]) (Table 2).

**Table 2.** Adrenocorticotropin stimulation test

Variable	0 min	30 min	60 min
Cortisol	0.28 µg/dL (SI: 7.72 nmol/L)	2.60 µg/dL (SI: 71.7 nmol/L)	3.46 µg/dL (SI: 95.4 nmol/L)
Adrenocorticotropin	<1.5 pg/mL	ND	ND

Abbreviations: ND, no data; SI, international system of units.

Subsequently, a CRH stimulation test was performed to evaluate the ability of the pituitary gland to secrete ACTH in response to CRH stimulation. Cortisol and ACTH levels were measured from blood samples collected at baseline and after 30, 60, and 90 minutes following IV administration of a 100-mcg CRH bolus. Cortisol levels peaked to 0.28 µg/dL (SI: 7.7 nmol/L) at 30 minutes (reference range, < 18 µg/dL), and ACTH levels were less than 1.5 pg/mL (SI: < 0.3303 pmol/L) (negative; reference range, <twice baseline) (Table 3).

**Table 3.** Corticotropin-releasing hormone test

Variable	0 min	30 min	60 min	90 min
Cortisol	0.31 µg/dL (SI: 8.55 nmol/L)	0.28 µg/dL (SI: 7.72 nmol/L)	0.24 µg/dL (SI: 6.62 nmol/L)	0.23 µg/dL (SI: 6.34 nmol/L)
Adrenocorticotropin	11.0 pg/mL	< 1.5 pg/mL	< 1.5 pg/mL	< 1.5 pg/mL

Abbreviation: SI, international system of units.

Despite the markedly low cortisol and ACTH response, other anterior pituitary hormones were within normal limits, and there were no clinical features indicating a broader pituitary dysfunction. Overall, the patient's clinical presentation was consistent with secondary adrenal insufficiency due to IAD.

## Treatment

Eight weeks after hospitalization, IV hydrocortisone therapy was initiated at a dose of 50 mg per day and was later transitioned to oral administration. Given the absence of hypotension or hyponatremia, this initial dose was selected with careful monitoring and readiness to increase if signs of adrenal crisis emerged. The dose was gradually tapered, and by 9 weeks after hospitalization, it was reduced to an oral maintenance dose of 15 mg daily, which was continued until discharge.

## Outcome and Follow-up

After hydrocortisone administration, the patient's level of consciousness improved. By 10 weeks after hospitalization, she was awake, alert, and oriented.

Three months after hospitalization, the patient was transferred to another hospital for rehabilitation to regain physical strength following prolonged bed rest. Her nausea, vomiting, and depressive symptoms resolved, and her appetite returned to normal.

## Discussion

IAD often presents with nonspecific neuropsychiatric or gastrointestinal symptoms, making its diagnosis challenging. Symptoms such as anorexia, nausea, fatigue, and depression are easily misinterpreted as primary psychiatric or gastrointestinal disorders [4]. In a systematic review, apathy (38%) was the most frequently reported symptom, followed by weight loss (25%), anorexia (22%), nausea or vomiting (8%), and cognitive impairment (8%) [5]. The present case followed a similar course, beginning with such nonspecific symptoms that eventually progressed to life-threatening adrenal insufficiency.

This case had several notable features. First, the patient initially developed depressive symptoms, which progressed to gastrointestinal complaints and, within a few weeks, culminated in severe adrenal insufficiency. Second, despite the absence of overt metabolic derangements such as hyponatremia or hypoglycemia and no evidence of structural brain lesions, the patient developed a marked disturbance of consciousness (unresponsiveness). Most previously reported cases have described either psychiatric or somatic symptoms alone, and only a small number of reports have documented severe multisystemic manifestations. Third, this case represented idiopathic IAD, without typical risk factors such as chronic glucocorticoid use, pituitary irradiation, or immune checkpoint inhibitor therapy. These findings highlight the importance of considering IAD in the differential diagnosis of rapidly progressive, multisystem symptoms, particularly in older patients [6].

Despite extensive evaluations by psychiatry, gastroenterology, and emergency medicine departments over several weeks, the underlying diagnosis remained elusive. The failure to recognize an endocrine etiology despite multidisciplinary involvement underscores a systematic diagnostic blind spot in the evaluation of IAD. Cognitive biases played a considerable role in this delay. Depression is a well-recognized manifestation of various endocrine disorders, including hypothyroidism, adrenal insufficiency, and diabetes mellitus [1]. In this case, the neuropsychiatric symptoms were initially attributed to primary depressive disorder, and antidepressant therapy was initiated. However, the patient's symptoms persisted and progressively worsened despite treatment. Anchoring bias—the fixation on the initial diagnosis—may have constrained further endocrine reasoning. This bias represents a major obstacle to accurate diagnosis when neuropsychiatric symptoms predominate. The possibility of IAD was raised only after hormonal testing was performed in response to worsening consciousness, underscoring the necessity of reevaluation when a patient fails to respond to standard therapy. Clinicians should consider endocrine disorders such as IAD in patients with depressive-like symptoms and altered consciousness, especially when the course is atypical or progressive.

Another key feature of this case was the absence of typical risk factors for IAD, including chronic glucocorticoid therapy, pituitary irradiation, or immune checkpoint inhibitor treatment. Although IAD is increasingly recognized in the context of immunotherapy-induced hypophysitis, truly idiopathic cases such as this remain relatively rare. The etiology of idiopathic IAD has not been fully elucidated, but an autoimmune mechanism involving antipituitary antibodies has been suggested. Patients without typical risk factors often experience diagnostic delays. Even in the absence of identifiable predisposing factors, IAD should be considered when patients present with unexplained multisystemic manifestations or treatment-resistant neuropsychiatric symptoms. This case illustrates that the absence of known triggers can further complicate diagnosis, emphasizing the importance of hormonal evaluation in patients with unexplained multiorgan dysfunction. In older patients, nonspecific neuropsychiatric or gastrointestinal symptoms are often attributed to age-related changes or common comorbidities, leading to missed or delayed diagnosis.

Adrenal insufficiency can cause impaired consciousness, typically in association with marked hyponatremia or hypoglycemia [7]. Notably, in this case, the patient developed severe neurological deterioration (unresponsiveness) despite only mild metabolic abnormalities. The unresponsive state improved dramatically following the initiation of hydrocortisone replacement, strongly suggesting cortisol deficiency–related encephalopathy. Cortisol is essential for maintaining central nervous system excitability, neurotransmitter regulation, and blood-brain barrier integrity; its deficiency can produce a wide spectrum of neurological symptoms, ranging from subtle cognitive impairment to life-threatening coma. Although cognitive changes in older patients with depression can be explained by catatonia or pseudodementia, the rapid improvement following hormonal replacement in this patient strongly supports a direct role of cortisol deficiency in her neurological dysfunction. This finding indicates severe consciousness disturbance can occur in IAD even in the absence of overt metabolic crises and that timely glucocorticoid replacement can fully restore neurological function.

This case demonstrates that IAD can mimic primary neuropsychiatric disorders and, if unrecognized, can progress to severe systemic complications. The prolonged diagnostic delay in this patient led to substantial physical deterioration, including necessitating total parenteral nutrition and rehabilitation. However, hydrocortisone therapy led to rapid neurological and systemic recovery. This clinical course indicates that IAD is a treatable cause of secondary adrenal insufficiency and that even patients presenting with profound neurological impairment can achieve clinical remission with timely glucocorticoid replacement. The present case highlights the potential for unrecognized endocrine etiologies in patients with neuropsychiatric presentations and underscores the need for comprehensive internal medicine evaluation. Therefore, in patients with unexplained neuropsychiatric or multisystemic symptoms—particularly those showing poor response to conventional therapies—IAD should be actively considered as an important differential diagnosis. In older patients, nonspecific symptoms may represent the initial manifestation of IAD. Because IAD is a potentially reversible disorder with favorable outcomes when detected early, timely diagnosis is crucial to prevent fatal adrenal crises and optimize functional prognosis.

## Learning Points

- IAD can present with nonspecific neuropsychiatric and/or gastrointestinal symptoms, leading to misdiagnosis, especially in older patients.
- Altered mental status in patients with apparent depression should be promptly evaluated for possible endocrine causes, particularly if the clinical course is atypical or deteriorating.
- Diagnostic delay is common when patients with IAD present without classic risk factors (eg, glucocorticoid use, pituitary irradiation, and immunotherapy), highlighting the importance of hormonal screening for unexplained multisystem symptoms.
- Anchoring bias can lead to prolonged misdiagnosis in cases in which neuropsychiatric symptoms dominate; reassessment is critical when standard treatments fail.
- IAD is a treatable cause of secondary adrenal insufficiency, and timely recognition and corticosteroid replacement can result in full clinical recovery even in cases of severe neurological deterioration.

## Data Availability

Original data generated and analyzed during this study are included in this published article.

## Abbreviations

ACTH: adrenocorticotropin
CRH: corticotropin-releasing hormone
CT: computed tomography
IAD: isolated adrenocorticotropin deficiency
IV: intravenous
MRI: magnetic resonance imaging
